# Quasiparticle interaction originating from Bogoliubov Fermi surfaces under pressure in 18%-S substituted FeSe studied via NMR

**DOI:** 10.1038/s41598-025-13717-6

**Published:** 2025-08-14

**Authors:** Zhongyu Yu, Xiaoling Shen, Koya Nakamura, Kazuya Inomata, Kohei Matsuura, Yuta Mizukami, Shigeru Kasahara, Yuji Matsuda, Takasada Shibauchi, Yoshiya Uwatoko, Naoki Fujiwara

**Affiliations:** 1 Graduate School of Human and Environmental Studies, Kyoto University, Yoshida-Nihonmatsu-cho, Sakyo-ku, Kyoto, 606-8501 Japan; 2 Institute for Solid State Physics, University of Tokyo, 5-1-5 Kashiwanoha, Kashiwa, 277-8581 Chiba Japan; 3 Department of Advanced Materials Science, University of Tokyo, 5-1-5 Kashiwanoha, Kashiwa, 277-8561 Chiba Japan; 4 Department of Physics, Kyoto University, Kitashirakawa Oiwake-cho, Sakyo-ku, Kyoto, 606-8502 Japan; 5 Present Address: Key Laboratory of Artificial Structures and Quantum Control, School of Physics and Astronomy, Shanghai Jiao Tong University, Shanghai, 200240 China; 6 Present Address: NEC Platforms, Ltd, 1753-1 Shimonumabe, Nakahara-ku, Kawasaki, 211-8666 Kanagawa Japan; 7 Present Address: DeNA Co., Ltd, 6-30-15 Hommachi, Shibuya-ku, Tokyo, 151-0071 Japan; 8 Present Address: Department of Applied Physics, University of Tokyo, 7-3-1 Hongo, Bunkyou-ku, Tokyo, 113-8656 Japan; 9 Present Address: Department of Physics, Graduate School of Science, Tohoku University, Sendai, 980-8578 Miyagi Japan; 10 Present Address: Research Institute for Interdisciplinary Science, Okayama University, Okayama, 700-8530 Japan

**Keywords:** Superconducting properties and materials, Magnetic properties and materials

## Abstract

S-substituted FeSe superconductors in the tetragonal phase display several unique features among iron-based superconductors, particularly the presence of zero-energy excitations in the superconducting (SC) state. The recent concept of Bogoliubov Fermi surfaces (BFSs)—a theoretical model describing ultranodal states—has attracted considerable interest. Nuclear magnetic resonance (NMR) studies on FeSe_1 − *x*_S_*x*_ (*x* = 0.18) have revealed an anomalous low-energy spin fluctuations deep in the SC state. The low-energy spin fluctuations are enhanced with decreasing temperature, supporting strong Bogoliubov quasiparticle interactions associated with BFSs. Here, we further investigate these correlation effects through ^77^Se-NMR measurements of FeSe_1 − *x*_S_*x*_ (*x* = 0.18) under pressures up to 2.0 GPa and temperatures down to ~ 100 mK. The results demonstrate that the anomalous enhancement is suppressed but persists under pressure, implying that quasiparticle interactions become weak by applying pressure. Furthermore, spin fluctuations in the normal state exhibit different temperature dependence from those deep in the SC state, suggesting that the nesting properties of normal electrons differ from those of Bogoliubov quasiparticles. These findings are consistent with the theoretical model of BFSs with *C*_2_ symmetry and strengthen evidence for Bogoliubov quasiparticle interactions, providing insights into the unconventional pairing state of this system.

**Supplementary Information:**

The online version contains supplementary material available at 10.1038/s41598-025-13717-6.

## Introduction

Iron selenide, FeSe is a unique superconductor among various iron-based superconductors in that it has extremely shallow unconnected Fermi surfaces (hole and electron pockets) and has presented fascinating physics related to superconductivity^1^. FeSe has unique phase diagrams: the superconductivity coexists with nematicity at ambient pressure without magnetism, while the superconductivity coexists with magnetism under pressure instead of nematicity^2^. Such unique features are maintained for S substitution to some extent^3^. At ambient pressure, the nematicity in FeSe_1 − *x*_S_*x*_ extends up to the nematic quantum critical point (QCP) (*x*_c_≃0.17)^4,5^. Superconducting (SC) transition temperature, *T*_c_ of pure FeSe (= 9 K) is maintained for S substitution up to *x*_c_ and decreases by half when crossing the QCP^6^.

Among a variety of topics associated with superconductivity, the topologically protected nodal planes, referred to as Bogoliubov Fermi surfaces (BFSs)^7,8^ have been paid much attention in both theoretical and experimental aspects for a heavily S-substituted regime over *x* > *x*_c_. BFSs are attainable in a multiband system under the condition of a strong spin-orbital coupling and time-reversal symmetry breaking (TRSB). An anomalous residual density of states (DOS) in the tetragonal phase with *C*_4_ symmetry has raised attention to the study of BFSs. Survival of large DOS in the SC state was found from specific heat^6,9^ and scanning tunneling spectroscopy (STS)^5,10^ measurements. Following these experimental results, C. Setty et al. have presented a theoretical model based on BFSs in the SC state^8,11^ and calculations derived from this model have reproduced these experimental results. The TRSB required for BFSs has been suggested by muon spin relaxation measurements^12^. In recent angular-resolved photoemission spectroscopy (ARPES) experiments^13^ heavily S-doped FeSe has exhibited wide nodal regions with *C*_2_ symmetry in the SC state, despite the tetragonal phase with *C*_4_ symmetry. Y. Cao et al. proposed a microscopic model that includes intraband spin-singlet pairing, interband nonunitary spin-triplet pairing, and ferromagnetic exchange coupling^14^. They found that such BFSs with *C*_2_ symmetry can be reproducible as well as other pairing symmetry by tuning gap parameters. Recent NMR studies on FeSe_1 − *x*_S_*x*_ (*x* = 0.18)^18^ have shown an unusual enhancement of low-energy spin fluctuations, namely an unusual upturn of the relaxation rate divided by temperature (1/*T*_1_*T*) as temperature decreases to nearly zero, which upholds the strong Bogoliubov quasiparticle interactions in addition to the presence of BFSs. In the follow-up theoretical paper, Y. Cao et al. calculated the spin susceptibilities for the ultranodal states in a minimal two-band model, where the interband nonunitary spin-triplet pairing is responsible for the BFSs^15^. They concluded that certain scattering between coherent spots/segments on the BFSs can get strongly enhanced, resulting in a robust upturn in the relaxation rate when the interaction is strong. Recently, impurity effect on BFSs has been studied using electron irradiation^16^.

To investigate the correlation effect deep in the SC state is of great importance not only for establishing the presence of BFSs but also for offering insights into the pairing symmetry of the system. In the present work, we performed ^77^Se-NMR to 100 mK under pressure up to 2.0 GPa using a NiCrAl pressure cell^17^.

## Experimental results

For the study on S-substituted FeSe, the SC state has been investigated at ambient pressure so far^18^ while the paramagnetic state was investigated up to several pressures by two NMR groups^19–23^. The SC state of pure FeSe was studied by several NMR works^24–27^. Many of them investigated how the superconductivity breaks under a very high field. In general, the relaxation rate divided by temperature 1/*T*_1_*T* provides a measure of low-energy spin fluctuations,

1 $$\frac{1}{{T}_{1}T}\sim\frac{1}{\omega\:}\sum\:_{\mathbf{q}}\text{I}\text{m}\chi\:\left(\mathbf{q}\right)$$

where *χ*(**q**) is the wave-number (**q**)-dependent susceptibility. For *x* = 0.18, we found an upturn of 1/*T*_1_*T* with decreasing temperature deep in the SC state at ambient pressure in the same manner as the upturn of 1/*T*_1_*T* toward *T*_c_ in the normal state^18^. The upturn of 1/*T*_1_*T* deep in the SC state is extremely rare in the SC compounds. It can hardly be explained by extrinsic effects such as the impurity effect^28^, Volovik effect^29^ or spatial inhomogeneity^5^. In the case of the Volovik effect, 1/*T*_1_*T* = constant should appear at low *T* below *T*_c_ as well as the impurity effect, and the constant value should depend on the magnetic field^29^. Experimentally, 1/*T*_1_*T* = constant is not observed for *x* = 0.18 but for *x* = 0.05 and 0.10. However, the value differs by one order of magnitude between them, despite that *T*_c_s are almost the same and the applied magnetic field was also the same. Such behavior rules out the possibility of the Volovik effect as well as the impurity effect. The upturn of 1/*T*_1_*T* below *T*_c_ is attributed to the scattering between segments on BFSs^15^ and gives evidence of the presence of BFSs as discussed in the following contents. To study how external parameters such as pressure influence quasiparticle interactions is of great importance to establish the presence of BFSs. We performed ^77^Se-NMR experiments under pressure and found that application of pressure up to 2.0 GPa weakens the scattering on BFSs.

**Fig. 1:**
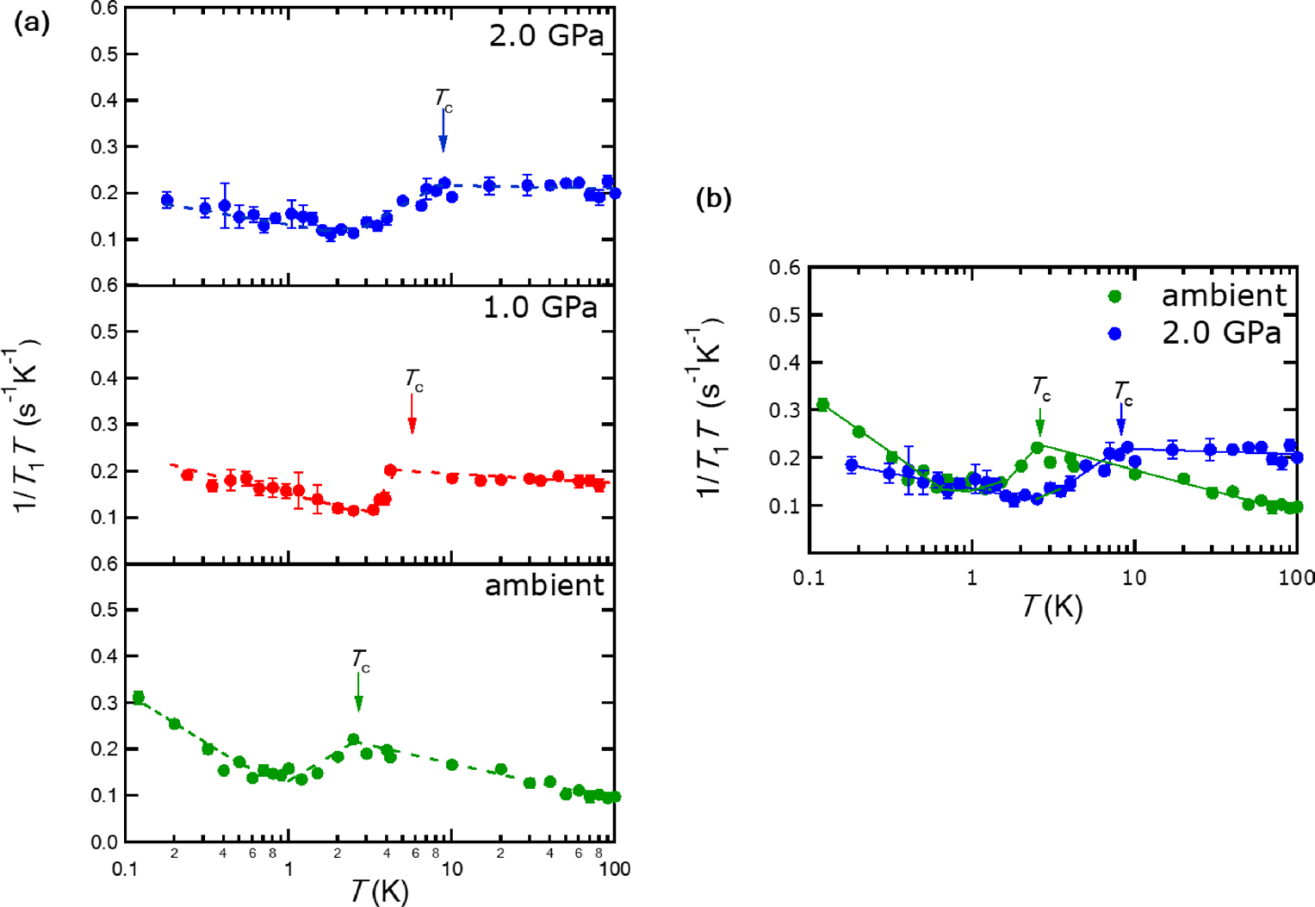
(**a**) Temperature (*T*) dependence of relaxation rate (1/*T*_1_) divided by *T*, 1/*T*_1_*T*, obtained from ^77^Se-NMR experiments at several pressure levels on a single crystal of FeSe_1 − *x*_S_*x*_ (*x* = 0.18). The magnetic field was applied parallel to the FeSe plane. Superconducting transition temperature *T*_c_ was measured at 6.02 T by AC susceptibility measurements using a network analyzer. Dashed lines are guides to the eye. (**b**) Comparison of 1/*T*_1_*T* at 2.0 GPa with that at ambient pressure. Solid lines are guides to the eye.

Figures 1a and 1b show an overview of 1/*T*_1_*T* under pressure, which is the main focus of this study. In the normal state, an upturn toward *T*_c_ is evident at ambient pressure, but it becomes ambiguous at 1.0 GPa, and 1/*T*_1_*T* becomes almost constant at 2.0 GPa. Interestingly, similar suppression of the upturn under pressure is also observed in the SC state below *T*_c_. We will discuss the suppression under pressure in the following section.

**Fig. 2:**
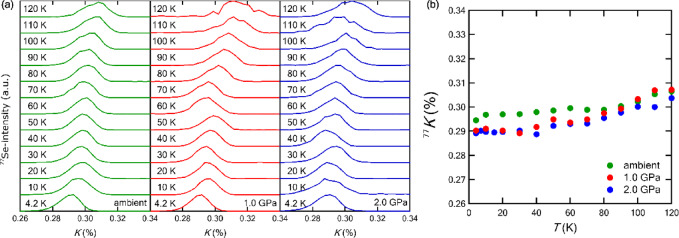
(**a**) NMR spectra for FeSe_1 − *x*_S_*x*_ (*x* = 0.18) at ambient pressure,1.0 GPa, and 2.0 GPa, resulting from the FFT of the spin-echo signal. (**b**) Knight shift (*K*) obtained from the peak positions of NMR spectra shown in Fig. 2a.

Figure 2a shows the NMR spectra at ambient pressure, 1.0 GPa, and 2.0 GPa, resulting from the FFT of the spin-echo signal. For all three pressure levels, double-peak structure corresponding to the nematic order^30^ was not observed throughout the measured temperature range, which is consistent with the results obtained by other techniques. The double-peak structure has been detected at the substitution level below *x*_c_ at ambient pressure. Furthermore, disappearance of the echo signal corresponding to AFM ordering was not observed for *x* = 0.18 up to 2.0 GPa in contrast to the case of *x* = 0.12 at 3.9 GP where AFM order appears^22^. These results indicate that FeSe_1 − *x*_S_*x*_ (*x* = 0.18) is located in the paramagnetic *C*_4_ state up to 2 GPa. Figure 2b shows the Knight shift (*K*) obtained from NMR spectra in Fig. 2a. In all three pressure levels, *K* decreases gently with decreasing temperature and becomes constant at lower temperature regions. Additionally, *K* also decreases slightly with increasing pressure. The tendency is consistent with that for *x* = 0.12 ^22^, 0.15, and 0.29^23^.

The Knight shift can be expressed as the sum of spin part and orbital part, i.e.

2 $$K={K}_{\text{s}\text{p}\text{i}\text{n}}+{K}_{\text{o}\text{r}\text{b}},$$

in which *K*_spin_ is proportional to the density of states (DOS) and the uniform spin susceptibility χ(0). At low temperatures, both *K*_*s*pin_ and *K*_orb_ are *T*-independent, and cannot be obtained independently from experiments. The two components are usually separated from each other by the theoretical estimation of *K*_orb_. The decrease of *K* due to pressure application should be attributed to that of *K*_orb_, since the DOS of two-dimensional electron systems is constant and proportional to *m/*ℏ ^22^, where *m* is the effective mass and ℏ is the Planck constant divided by 2π, and consequently, *K*_spin_ should be constant.

## Discussion

### Upturn of 1/*T*_1_*T* at ambient pressure (*T* > *T*_c_)

**Fig. 3:**
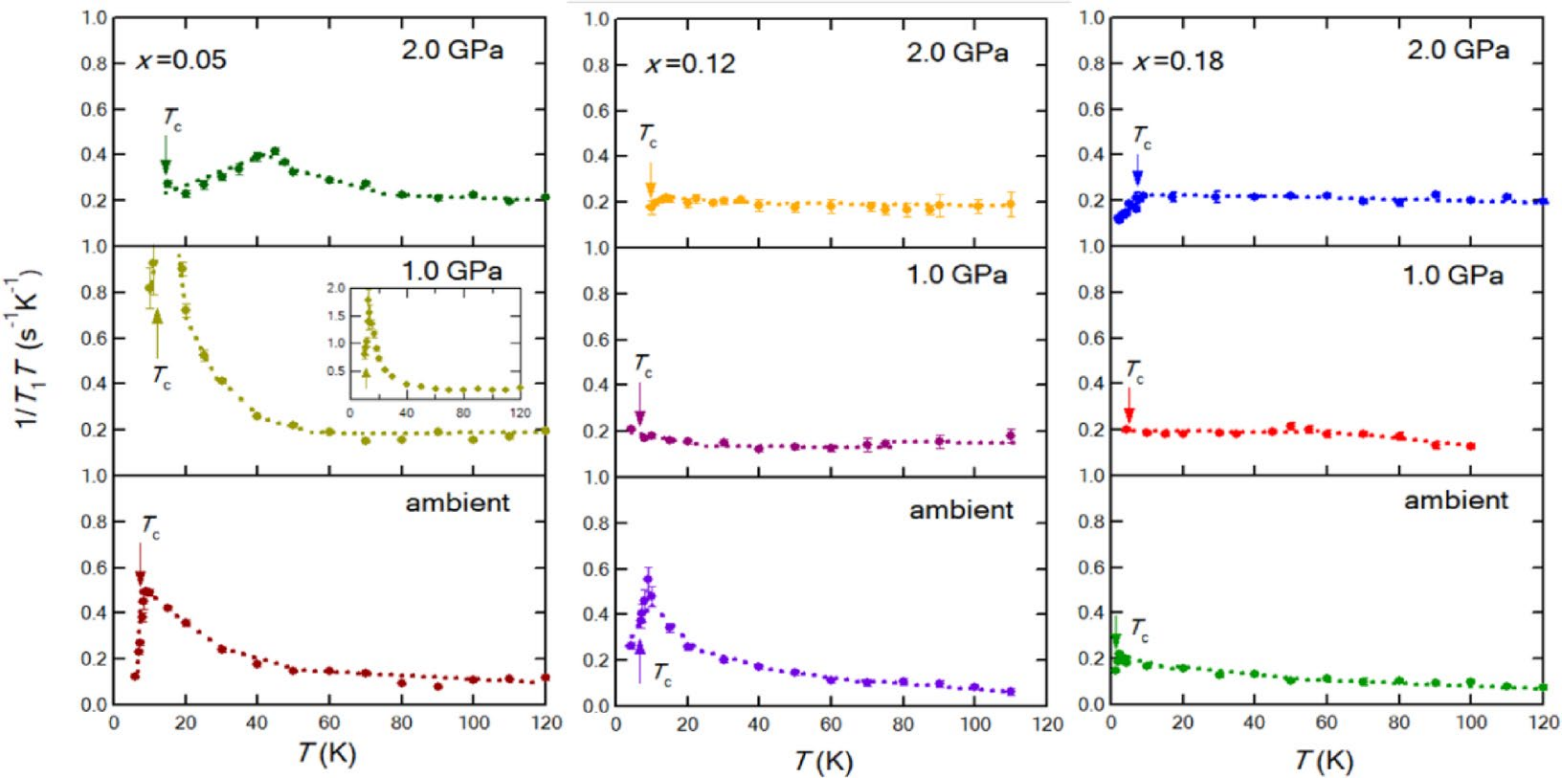
An overview of 1/*T*_1_*T* for three different substitution levels, *x* = 0.05, 0.12 ^20^, 0.18, crossing the nematic QCP. The data of the inset is the same with those of the main panel but displayed in different scale.

**Fig. 4:**
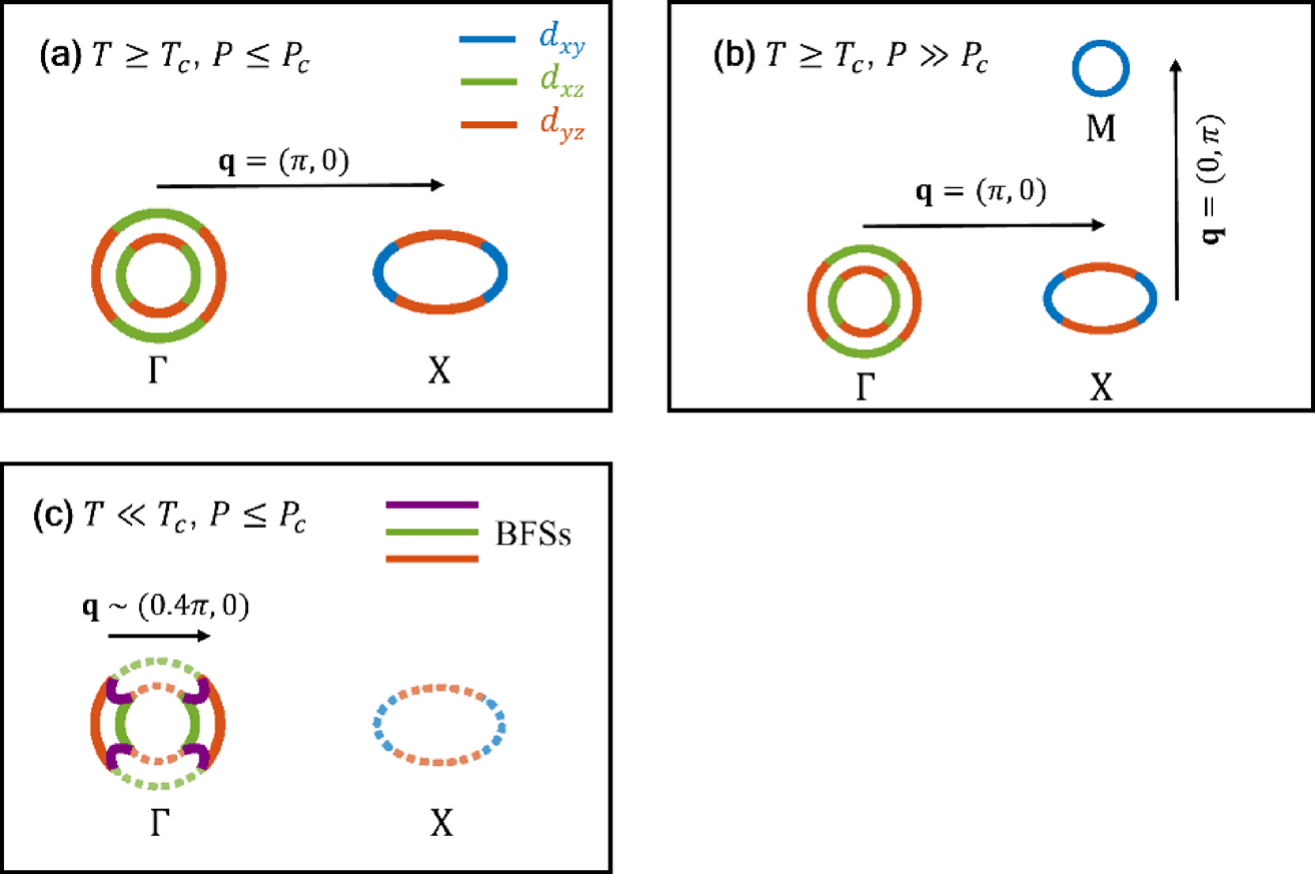
(**a**) Original Fermi surfaces (OFSs) in the normal state at ambient pressure. (**b**) OFSs in the normal state under pressure with another hole pocket emerging at point M, $\:\varvec{k}=(\pi\:,\pi\:)$. (**c**) Bogoliubov Fermi surfaces (BFSs) in the SC state^15^. OFSs in the normal metallic state are shown by dashed curves for comparison. BFSs in the superconducting state are plotted by solid curves. The segments colored in red and green represent BFSs with the character of small intraband spin-singlet particle-hole mixing, and the segments colored in purple represent BFSs with the character of significant interband spin-triplet particle-hole mixing.

The upturn of 1/*T*_1_*T* toward *T*_c_ in the normal state originates from spin fluctuations with **q** ≠ 0, as seen from the comparison of *T* dependence between *K*_spin_ and 1/*T*_1_*T*. Similarly to iron-based pnictides, spin fluctuations are associated with the topological configuration of electron and hole pockets and interband coupling. The enhancement of *χ*(**q**) is expected at **q** ~ (π, 0) in the nematic phase as well as in the tetragonal phase as shown in Fig. 3, owing to the interband coupling between electron and hole pockets, namely the **q** = (π, 0) nesting, as shown in Fig. 4(a). The enhancement of χ(**q**) at **q** ~ (π, 0) was observed for pure FeSe and 7%-S substituted FeSe in neutron inelastic scattering measurements^31–34^.

### Pressure-induced Lifshitz transition

The upturn of 1/*T*_1_*T* above *T*_c_ is suppressed with increasing pressure, and 1/*T*_1_*T* becomes a constant at 2.0 GPa. The behavior is not specific to *x* = 0.18 but is common to all substitution levels. At a high-pressure region, a pressure-induced Lifshitz transition occurs as suggested from both theoretical and experimental aspects^20,22,35–38^. Figure 3 gives an overview of three different substitution levels *x* = 0.05, 0.12^22^, and 0.18, crossing the nematic QCP. The data for *x* = 0.18 is the same as those shown in Figs. 1a and 1b, although the upturn at ambient pressure looks small on this scale. Similar behavior has also been observed by another NMR study for *x* = 0.20, and Fermi-liquid behavior was suggested in their study^23^.

According to the theoretical investigation^35^reconstruction of the Fermi surfaces or predominant **q**, namely Lifshitz transition, occurs at around 2 GPa due to the emergence of *d*_xy_ hole pocket at ***k***~(π, π) as shown in Fig. 4b, which could cause the **q**=(0, π) nesting between electron and hole pockets at a high pressure regime, as expected in the *P*-*T* phase diagram of Fe(Se, S)^3^. At the crossover region where the dominant **q** reconstructs and the nesting changes, spin fluctuations become tentatively *T*-independent before forming a new nesting condition.

Notably, an extremely strong peak is observed for *x* = 0.05 at 1.0 GPa. However, it is strongly suppressed at 2.0 GPa, and another peak corresponding to AFM ordering appears at around 40 K. This phenomenon is explained by the Lifshitz transition occurring at a lower pressure region below 2 GPa. The linewidth broadens a little at around 40 K. For *x* = 0.12, the AFM ordering is detected at a higher pressure as the loss of signal^22^as shown in Supplementary Fig. 2.

### Spin correlation at 2.0 GPa (*T* > *T*_c_)

The suppression of the upturn under pressure implies that the interaction between electrons becomes weak under pressure. The interaction at 2.0 GPa can be estimated given that 1/*T*_1_*T* is almost *T*-independent and obeys the Korringa relation:

3 $$\frac{1}{{T}_{1}T}=\frac{4\pi\:{k}_{\text{B}}}{\hslash\:}\:{\left(\frac{{\gamma\:}_{n}}{{\gamma\:}_{e}}\right)}^{2}{K}_{\text{s}\text{p}\text{i}\text{n}}^{2}K\left(\alpha\:\right).$$

The interaction *U* is included in *α* as *α* = *U*$\:{\chi\:}_{0}$where $\:{\chi\:}_{0}$ is the uniform susceptibility of free electrons. *K*(*α*) provides a measure of electron correlation^18^:

4 $$K\left(\alpha\:\right)=\:\:\frac{{\left(1\:-\:\alpha\:\right)}^{2}}{{\langle{\left(1-\frac{\alpha\:{\chi\:}_{0}\left(\mathbf{q}\right)}{{\chi\:}_{0}}\right)}^{2}\rangle}_{\text{F}\text{S}}},$$

where $\:{\langle\:{\left(1-\frac{\alpha\:{\chi\:}_{0}\left(q\right)}{{\chi\:}_{0}}\right)}^{2}\rangle\:}_{FS}$ represents the average over **q** connecting two points on the Fermi surfaces, and $\:{\chi\:}_{0}\left(\mathbf{q}\right)$ is the **q**-dependent susceptibility without the interaction. For ferromagnetic metals, *K*(*α*) < 1, whereas for antiferromagnetic metals, *K*(*α*) > 1. For free electrons, *K*(*α*) = 1 and *α* = 0. For pure FeSe, *K*_orb_≃0.23% and *K*_spin_=0.06% were estimated from the *K*-χ plot^39^while *K*_orb_/*K*_spin_≃5 estimated from RPA calculations^40^ leads to *K*_orb_≃0.25% and *K*_spin_=0.05%. For *x* = 0.12, *K*_orb_≃0.26% and *K*_spin_= 0.03% were estimated from the *K-*χ plot^22^. These results imply that *K* is almost insensitive to S-substitution levels and is estimated to be approximately 0.3% for *x* up to ~ 0.29 ^23^. As for pressure dependence, *K* decreases only 0.01% by applying pressure of 2.0 GPa, as shown in Fig. 2b. The decrease is approximately the same for *x* = 0.12, implying that *K* is also almost insensitive to pressure. We estimate *K*(*α*)≃15 from 1/*T*_1_*T*≃0.21 assuming *K*_spin_=0.03% at 2.0 GPa for *x* = 0.18. The results suggest that AFM fluctuations still remain even at 2.0 GPa, although AFM fluctuations or the upturn toward *T*_c_ is strongly suppressed with increasing pressure.

### Theoretical model based on BFSs with *C*_2_ symmetry (*T* < *T*_c_)

Similarly to the normal state, we suggested that 1/*T*_1_*T* deep in the SC state would be explained by a nesting scenario because the upturn below *T*_c_ resembles the upturn above *T*_c_^18^ In this nesting scenario, BFSs are supposed to exist for both hole and electron pockets. However, it is unclear whether BFSs exist on the electron pocket at the present stage, because the gap closing has been observed only for a hole pocket at Γ point from the Laser ARPES measurements^13^.

Contrary to the scenario above, the recent theoretical investigation^15^ pointed out that the upturn below *T*_c_ is explainable only by BFSs appearing at hole pockets as shown in Fig. 4c. This theoretical model assumes two hole pockets and BFSs with *C*_2_ symmetry at Γ point. The wave number **q**$\:\sim$(0.4π, 0) corresponding to a span between two BFSs segments contributes to an enhancement of *χ*(**q**) under the strong Hubbard-type interaction *U*. The BFSs segments colored in purple have the character of significant interband spin-triplet particle-hole mixing and moderate intraband spin-singlet particle-hole mixing. 1/*T*_1_*T* was calculated under the random phase approximation (RPA) for two cases where ***B*** is applied parallel (//) and perpendicular ($\:\perp\:$) to the quantization axis (*z*-axis) of the spin-triplet^15^. In both cases, the upturn of 1/*T*_1_*T* is reproduced. The case for ***B***$\:\perp\:$*z* causes a significant upturn at low temperatures and seems to reproduce the experimental results better than that for ***B***//*z* ^16^.

The upturn of 1/*T*_1_*T* originates from two factors: the nesting between BFSs segments and the interaction *U*. However, it is difficult to specify which contribution is larger from the experiments alone. According to the theoretical calculation of 1*/T*_1_*T* under RPA mentioned above^15^, the upturn is hardly expected if *U* is zero. This fact suggests that the upturn is sensitive to *U*. As shown in Fig. 1a and 1b the upturn of 1/*T*_1_*T* below *T*_c_ is suppressed at 2.0 GPa compared to that at ambient pressure, implying that *U* becomes weak but nonzero under pressure. The result seems reasonable considering *K*(*α*) ≃15 at 2.0 GPa in the normal state because *K*(*α*) ≃1 is expected for *U* = 0. The theoretical model would also explain why the *T* dependence of 1/*T*_1_*T* is different across *T*_c_: this is clearly seen from the data at 2.0 GPa where 1/*T*_1_*T* = constant above *T*_c_ whereas 1/*T*_1_*T* exhibits an upturn below *T*_c_. This change would be because the nesting properties change across *T*_c_. The predominant **q** would change from **q** = (π, 0) above *T*_c_ to **q**$\:\sim$(0.4π, 0) below *T*_c_, and $\:{\chi\:}_{0}$(**q**) in the SC state would be different from that determined by the nesting between normal electrons.

Recently, another theoretical model based on the pairing mediated by soft charge-nematic fluctuations has been proposed^41,42^. In this model, the strength depends on the location of a fermion along the Fermi surface, which induces nodal areas below *T* < *T*_c_. Our experimental results exhibit the upturn of 1/*T*_1_*T* down to T≃0.02*T*_c_. It is a future problem whether this model can reproduce the upturn of 1/*T*_1_*T* and its suppression under pressure.

## Conclusion

We have demonstrated that the enhancement of *χ*(**q**), namely the upturn of 1*/T*_1_*T*, at ambient pressure observed deep in the SC state is suppressed by the application of pressure, which implies that the scattering between the segments on nodal areas becomes weak but nonzero under pressure. The correlation on the nodal areas below *T*_c_ seems stronger than that between electron and hole pockets above *T*_c_, considering the results at 2.0 GPa. These experimental results are explainable by the recent theoretical model of BFSs with *C*_2_ symmetry at Γ point. The upturn of 1*/T*_1_*T* at ambient pressure is attributed to the enhancement of *χ*(**q**) under strong interaction at **q**≃(0.4π, 0), a span between two BFSs segments with the character of significant interband spin-triplet particle-hole mixing. These experimental facts contribute to establish the presence of the Bogoliubov quasiparticle interactions along with the presence of BFSs themselves. This study advances the understanding of interactions on the BFSs and offers a crucial step towards unraveling the nature of the pairing symmetry of the system.

## Methods

In this study, ^77^Se-NMR measurements of 18% S-substituted FeSe were conducted under hydrostatic pressure up to 2.0 GPa down to 100 mK. We used the sole single crystal with a size of 1.0 × 1.0 × 0.5 mm^3^ that was used in the previous measurements^18^. Nuclear magnetic relaxation time (*T*_1_) was measured by using the saturation-recovery method and evaluated by the exponential fitting of the recovery curve. Knight shift (*K*) was determined from the peak frequency of the fast Fourier transform (FFT) of the NMR signal. A magnetic field of 6.02 T was applied parallel to the FeSe plane (***B***//ab) to avoid vortices. We used a dilution refrigerator made by Bluefors for measurements at low temperatures below 1 K. Temperature fluctuations were suppressed within an accuracy of 0.01 K by PID control. The heating due to the NMR pulses was not observed within the experimental accuracy. These experimental conditions are the same with Ref^18^. To perform NMR measurements under pressure, we used a NiCrAl piston-cylinder pressure cell^17^ and monitored the pressure inside by ruby fluorescence measurements. For this purpose, we inserted an optical fiber with ruby powders on one end into the pressure cell together with an NMR coil. *T*_c_ was determined from AC susceptibility measurements utilizing the NMR tank circuit. The results measured at zero field and 6.02 T were shown in Supplementary Fig. 1.

## Supplementary Information

Below is the link to the electronic supplementary material.

Supplementary Material 1

## Data Availability

The data that support the findings of this study are available from the corresponding author upon reasonable request.
