# Supplementary material for: Quasiparticle interaction originating from Bogoliubov Fermi surfaces under pressure in 18%-S substituted FeSe studied via NMR
Source: Sci Rep. 2025 Aug 14;15:29824. doi: 10.1038/s41598-025-13717-6 (PMC12354710; doi:10.1038/s41598-025-13717-6)
Supplement: Supplementary file 1 — Supplementary Material 1 [file 41598_2025_13717_MOESM1_ESM.docx]

**Supplementary Material-Quasiparticle interaction originating from Bogoliubov Fermi Surfaces under pressure in 18%-S substituted FeSe studied via NMR**

Zhongyu Yu^1^, Xiaoling Shen^2,*^, Koya Nakamura^1,†^, Kazuya Inomata^1,§^, Kohei Matsuura^3,‡^, Yuta Mizukami^3,**^, Shigeru Kasahara^4,††^, Yuji Matsuda^4^, Takasada Shibauchi^3^, Yoshiya Uwatoko^2^, and Naoki Fujiwara^1,§§^

^1^Graduate School of Human and Environmental Studies, Kyoto University, Yoshida-Nihonmatsu-cho, Sakyo-ku, Kyoto, 606-8501, Japan

^2^Institute for Solid State Physics, University of Tokyo, 5-1-5 Kashiwanoha, Kashiwa, 277-8581, Chiba, Japan

^3^Department of Advanced Materials Science, University of Tokyo, 5-1-5 Kashiwanoha, Kashiwa, 277-8561, Chiba, Japan

^4^Department of Physics, Kyoto University, Kitashirakawa Oiwake-cho, Sakyo-ku, Kyoto, 606-8502, Japan

*Present address: Key Laboratory of Artificial Structures and Quantum Control, School of Physics and Astronomy, Shanghai Jiao Tong University, Shanghai 200240, China

^†^Present address: NEC Platforms, Ltd., 1753-1 Shimonumabe, Nakahara-ku, Kawasaki, Kanagawa, 211-8666, Japan

^§^Present address: DeNA Co., Ltd., 6-30-15 Hommachi, Shibuya-ku, Tokyo, 151-0071, Japan

^‡^Present address: Department of Applied Physics, University of Tokyo, 7-3-1 Hongo, Bunkyou-ku, Tokyo, 113-8656, Japan

^**^Present address: Graduate School of Science, Department of Physics, Tohoku University, Sendai, Miyagi, 980-8578, Japan

^††^Present address: Research Institute for Interdisciplinary Science, Okayama University, Okayama, 700-8530, Japan

^§§^Contact author: fujiwara.naoki.7e@kyoto-u.ac.jp

1. **DETERMINATION OF** $\boldsymbol{T}_{\boldsymbol{c}}$ **FROM THE AC SUSCEPTIBILITY**

The AC susceptibility measurements for *x*=0.18 were conducted to determine *T*_c_ by using the tank circuit attached to the head of an NMR probe at zero field and 6.02T. The magnetic field was applied parallel to the FeSe plane (***B***//ab) similarly to the *T*_1_ measurements.

The resonance frequency of the tank circuit (*f_r_*) was measured using a commercially available network analyzer. The frequency *f_r_* is related to the AC susceptibility χ as $f_{r}=1/\sqrt{LC(1+4\pi\chi)}$, where *C* and *L* represent the capacitance of a variable capacitor and the inductance of a coil wound onto the sample, respectively. The frequency *f_r_* increases gradually with decreasing temperature due to a gradual decrease in *L* during the cooling process, and changes drastically at *T*_c_ owing to the Meissner effect. We determined *T*_c_s from the crossing points of the dashed lines. *T*_c_s at zero field were 4.0, 6.7 and 12.4 K at ambient pressure, 1.0 and 2.0 GPa, respectively, while those at 6.02 T were 2.8, 6.1 and 9.5 K at ambient pressure, 1.0 and 2.0 GPa, respectively.


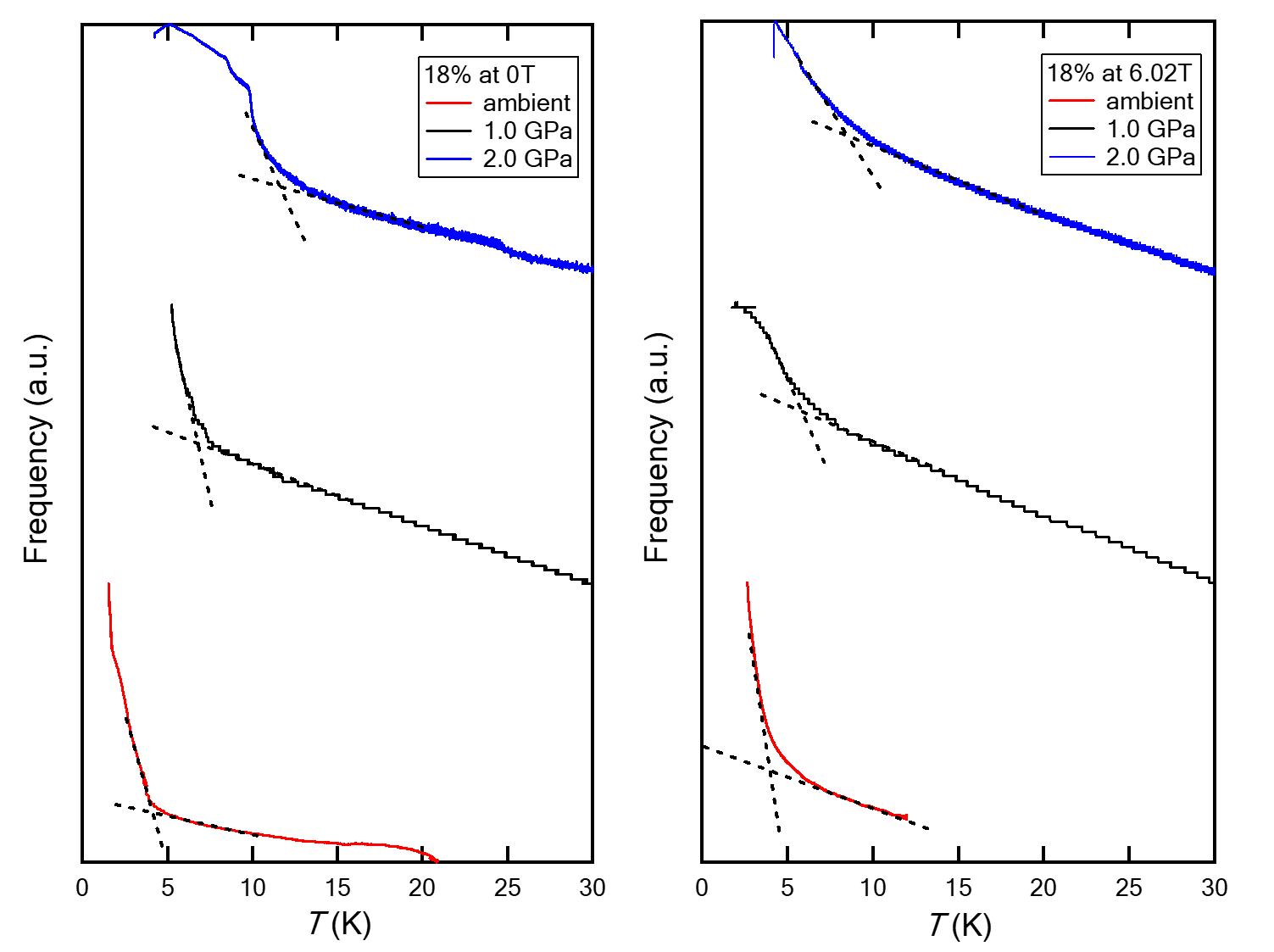


Figure 1: AC susceptibility measurements for FeSe_1-_*_x_*S*_x_* (*x*=0.18) using the tank circuit attached on the top of an NMR probe. *T*_c_s were determined from the crosspoints of dashes lines. Left and right panels represent the measurements at zero field and 6.02 T, respectively.

1. **EVIDENCE OF ANTIFERROMAGNETIC ORDER UNDER PRESSURE**

The appearance of antiferromagnetic (AFM) ordering can be observed from anomaly in linewidth of NMR spectra. Figure 2 shows the linewidth of *x* = 0.05 and 0.12 [1] at different pressure levels. For *x* = 0.12, NMR signals disappear as AFM order appears at 3.9 GPa, leading to an increase of linewidth, whereas no significant behavior is observed at 3.5 GPa where AFM order is absent. For *x* = 0.05, a hump is observed at 2.0 GPa and developes into a larger peak with further applying pressure to 2.8 GPa. The hump and the peak are considered as a precursor of AFM ordering and the intermediate stage of the development of AFM order, respectively. The NMR signal for *x*=0.05 is lost at *T*_c_ accompanied with the increase in linewidth.


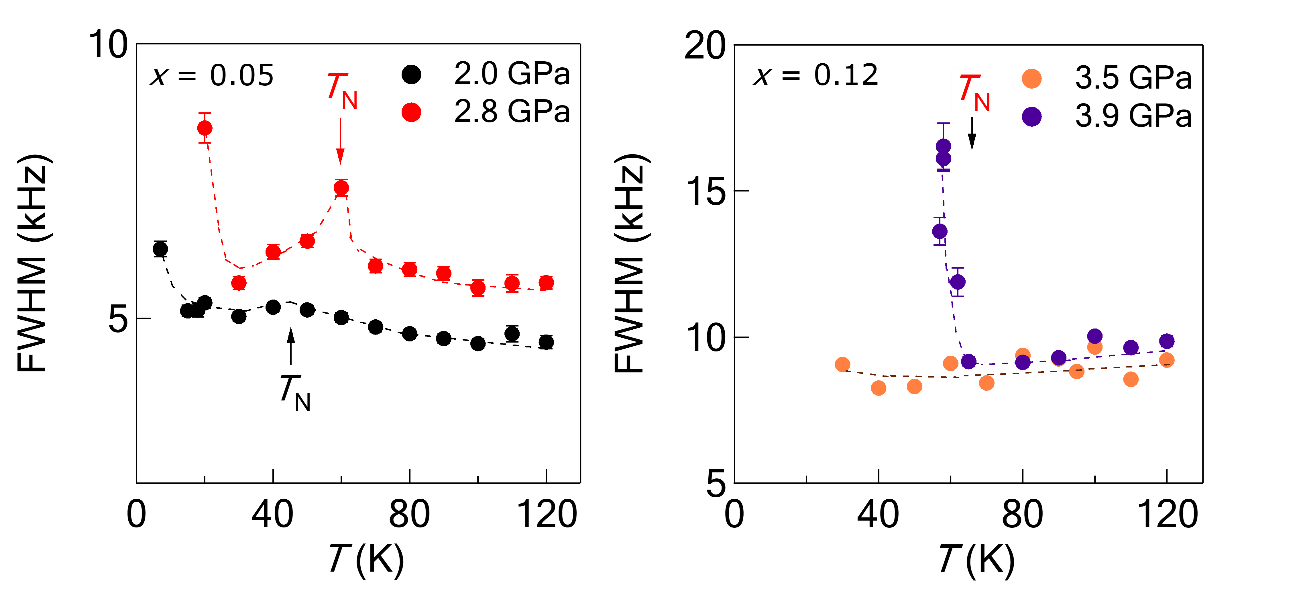


Figure 2: Linewidth of NMR spectra at different pressure levels for *x* = 0.05 and 0.12.

[1] T. Kuwayama, K. Matsuura, Y. Mizukami, S. Kasahara, Y. Matsuda, T. Shibauchi, Y. Uwatoko, and N. Fujiwara, ^77^Se-NMR study under pressure on 12%-S doped FeSe, J. Phys. Soc. Jpn. **88**, 033703 (2019).
